# Left ventricular assist device implants in patients on extracorporeal membrane oxygenation: do we need cardiopulmonary bypass?

**DOI:** 10.1093/icvts/ivab311

**Published:** 2021-11-11

**Authors:** Federico Pappalardo, Evgenij Potapov, Antonio Loforte, Michiel Morshuis, David Schibilsky, Daniel Zimpfer, Julia Riebandt, Christian Etz, Matteo Attisani, Mauro Rinaldi, Assad Haneya, Faiz Ramjankhan, Dirk Donker, Ulrich P Jorde, Daniel Lewin, Radi Wieloch, Rafael Ayala, Jochen Cremer, Letizia Bertoldi, Michael Borger, Artur Lichtenberg, Jan Gummert, Diyar Saeed, the Durable MCS after ECLS study group

**Affiliations:** 1 Advanced Heart Failure and Mechanical Circulatory Support Program, Vita-Salute San Raffaele University, Milan, Italy; 2 Department of Cardiothoracic and Vascular Anesthesia and Intensive Care, AO SS. Antonio e Biagio e Cesare Arrigo, Alessandria, Italy; 3 Department of Cardiac Surgery, German Heart Center Berlin, Berlin, Germany; 4 Department of Cardiac Surgery, Bologna University, Bologna, Italy; 5 Department of Cardiovascular and Thoracic Surgery, Heart and Diabetes Center NRW, Bad Oeynhausen, Germany; 6 Department of Cardiac and Vascular Surgery, Faculty of Medicine Freiburg University, Freiburg, Germany; 7 Department of Cardiac Surgery, Medical University Vienna, Vienna, Austria; 8 Department of Cardiac Surgery, Leipzig Heart Center, Leipzig, Germany; 9 Department of Cardiac Surgery, University of Turin, Turin, Italy; 10 Department of Cardiac Surgery, University Hospital Schleswig Holstein, Campus Kiel, Germany; 11 Department of Cardiothoracic Surgery, University Medical Center Utrecht, Utrecht, The Netherlands; 12 Department of Medicine, Montefiore Medical Center, Bronx, NY, USA; 13 CardioCenter, IRCCS Humanitas Clinical and Research Hospital, Rozzano, Italy; 14 Clinic for Cardiovascular Surgery, Dusseldorf University Hospital, Dusseldorf, Germany

**Keywords:** ECLS, CPB, assist device, outcome, mechanical circulatory support

## Abstract

**OBJECTIVES:**

Implanting a durable left ventricular assist device (LVAD) in a patient on extracorporeal life support (ECLS) is challenging. The goal of this study was to compare the results of patients from a European registry who had a durable LVAD implanted with or without transition from ECLS to cardiopulmonary bypass (CPB).

**METHODS:**

A total of 531 patients on ECLS support who had an LVAD implant between January 2010 and August 2018 were analysed; after 1:1 propensity score matching, we identified and compared 175 patients in each group.

**RESULTS:**

The duration of preoperative ECLS was 7 [standard deviation (SD) 6] vs 7 (SD 6) days in patients with or without CPB (*P* = 0.984). The surgical time was longer in the CPB group [285 (SD 72) vs 209 [SD 75] min; *P* ≤ 0.001). The postoperative chest tube output was comparable [1513 (SD 1311) vs 1390 (SD 1121) ml; *P* = 0.3]. However, re-exploration for bleeding was necessary in 41% vs 29% of patients with or without CPB (*P* = 0.01) and a significantly higher number of packed red blood cells and fresh frozen plasma [8 (SD 8) vs 6 (SD 4) units; *P* = 0.001 and 6 (SD 7) vs 5 (SD 5) units; *P* = 0.03] were administered to patients operated on with CPB. A postoperative mechanical right ventricular support device was necessary in 50% vs 41% of patients (*P* = 0.08). The stroke rate was not significantly different (*P* 0.99). No difference in survival was observed.

**CONCLUSIONS:**

Omitting CPB for an LVAD implant in patients on ECLS is safe and results in shorter operating time, less re-exploration for bleeding and fewer blood products. However, no survival benefit is observed.

**Figure:**
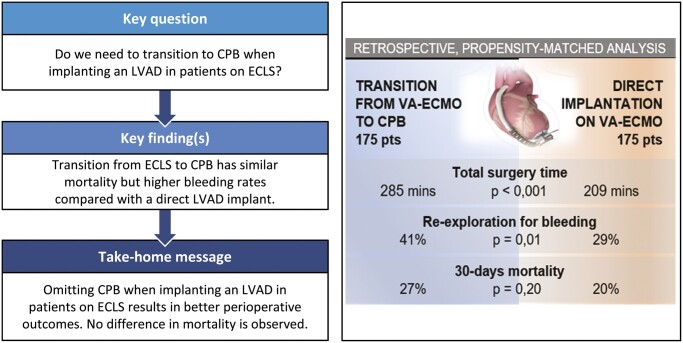

## INTRODUCTION

Continuous flow left ventricular assist devices (LVADs) have revolutionized the outcome of patients suffering from refractory heart failure [1, 2]; yet, this therapy is also applied ‘in extremis’ for patients with cardiogenic shock and temporary circulatory support. Indeed, implanting a durable LVAD in patients supported with extracorporeal life support (ECLS) is challenging, and all efforts should be made to decrease perioperative morbidity and mortality. Our group recently published the limited outcomes and postoperative morbidities of patients bridged with ECLS prior to durable mechanical circulatory support (MCS) [3].

With the goal of minimizing surgical trauma at the time of durable MCS implantation, several surgeons omit cardiopulmonary bypass (CPB) at the time of implantation [4]. It is unclear if omitting CPB would provide any clinical benefit for this critically ill patient population [5, 6]. CPB bears an inherent risk of coagulopathy and inflammatory response driven by the interaction of blood with air and the active collection of activated shed blood in the cardiotomy reservoir. On the other hand, CPB allows safe inspection of the left ventricular (LV) cavity and, eventually, thrombus removal when the LVAD is implanted. Understanding the challenges and the impact on outcomes in a large ‘real-world’ cohort of patients is paramount. The goal of this study was to compare the perioperative results of patients who had an LVAD implanted with or without switching from ECLS to CPB in the largest European registry (Durable MCS after ECLS registry).

## METHODS

### Ethical statement

Each institutional review board/ethical committee approved the study.

### Patient population

Our study represents a retrospective analysis; process and analysis were performed after approval from the institutional review board of each participating centre. The Durable MCS after ECLS registry is a multicentre retrospective study that gathered data on consecutive patients who had durable MCS implants while on ECLS between January 2010 and August 2018 in 11 European centres: Patients did not meet weaning criteria and were neurologically intact. A total of 531 patients were collected; patients requiring primary biventricular support with the SynCardia Total Artificial Heart (SynCardia, Tucson, AZ, USA) or a biventricular assist device, those who were implanted with a pulsatile LVAD and those who required concomitant procedures at the time of durable MCS implantation were excluded from this analysis.

Pre-, intra- and postoperative data were recorded. ECLS devices were implanted on an emergency basis; patients with postcardiotomy cardiogenic shock were also included. Weaning attempts under flow reduction and echocardiographic evaluation were performed in all patients according to institutional protocols. All preimplant (prior to durable MCS) patient characteristics, including renal and liver function tests (model of end-stage liver disease score), complete blood count and blood gas analysis parameters and inotropic support on the day the durable MCS was implanted, were evaluated. Long-term follow-up data were obtained from subsequent clinic visits. The Interagency Registry for Mechanically Assisted Circulatory Support definitions were used for postoperative complications.

The primary end-point was 30-day and long-term survival; postoperative bleeding (need for blood products and surgical re-exploration) as well as end organ dysfunction/failure including right ventricular failure, stroke and infections were considered as secondary outcome measures.

### Surgical procedures

At the time of the operation, intravenous heparin was administered with a target activated clotting time >400 s in all patients. A cell saver was used in both groups. In the CPB group, the ECLS cannulas were connected to the CPB machine during a short pump stop. The pump was implanted in a standard fashion using a full sternotomy in the CPB group; a less invasive approach (small anterolateral thoracotomy and j-sternotomy) was also used. The outflow graft was anastomosed to the ascending aorta after application of a side clamp. De-airing was performed through a needle inserted in the aorta and outflow graft when ECLS was used for surgery. Cannulas were removed at the end of the procedure, and full heparin reversal with protamine was administered. Blood products and coagulation factors were administered according to institutional guidelines. In case of right ventricular failure, mechanical right ventricular support was used. A temporary right ventricular assist device was implanted in the majority of the patients. In a few cases, the ECLS was left in place as mechanical right ventricular support. Anticoagulation was started in the intensive care unit as soon as bleeding (chest tube output and need for blood products) subsided. Patients requiring concomitant procedures were all operated on with CPB except for those having coronary artery bypass grafting.

Notably, the decision to proceed with the operation using CPB or ECLS was left to the surgeon’s preference; presumably, there might be institutional and surgeon bias across the 11 centres on which technique was selected. The presence of LV thrombus was considered a contraindication for omitting CPB. In these cases, CPB was used to remove thrombi prior to implanting the durable MCS.

### Statistical analyses

All statistical analyses were performed with SPSS for Windows (Version 22, 2013; IBM Corporation, Armonk, NY, USA). Demographic and clinical patient characteristics of both groups were compared with the independent samples Student’s *t*-test for continuous variables and the *χ*^2^ test for categorical variables. Group differences in intraoperative and postoperative variables were evaluated with the same method. The Fisher exact test was used for comparisons of categorical variables with a minimum expected cell count of 5 or less in 20% of cases. Survival was estimated using the Kaplan–Meier method. The log-rank test was used to compare survival differences between the groups and the stroke rate throughout the duration of support. Propensity score analysis was performed to identify 2 identical groups, 1 which was operated on without CPB (no CPB group) and 1 which was operated on using CPB (CPB group). Baseline criteria identified as clinically meaningful were entered for matching. Only this matched cohort was used for outcome analysis. Propensity scores were computed by binary logistic regression with CPB usage as an outcome variable. A 1:1 nearest neighbour matching algorithm with a calliper of <0.2 of the standard deviation of the logit of the propensity score was chosen to achieve the highest possible representativeness and precision. To check for residual imbalance of the propensity score matching (PSM) model, we used standardized mean differences for each matched variable. Due to the heterogeneous nature and number of clinical variables included in the matching process, we deemed a standardized bias threshold of 0.2 acceptable, also taking into account the perceived clinical impact of the respective variables. Clinical outcomes and differences between matched CPB and no-CPB groups were compared with the Student’s *t*-test for continuous variables and the *χ*^2^ test (or the Fisher exact test as appropriate) for categorical variables. A *P*-value of 0.05 was considered statistically significant.

## RESULTS

### Patient characteristics

Between January 2010 and August 2018, a total of 505 patients had durable MCS implanted while on ECLS because of failure to wean. Of these, 299 patients were operated on with conversion to CPB and 206 were left on ECLS; 93 patients who required concomitant procedures were excluded from the study. After 1:1 propensity matching, 175 patients were identified for each group and analysed. The baseline characteristics and the clinical presentation criteria were well balanced between the matched groups; detailed preoperative clinical characteristics are presented in Tables 1 and 2. The mean age of the patients in the CPB versus the ECLS group was 53 [standard deviation (SD) 11] and 52 (SD 11) years (*P* = 0.88). About 30% of the patients were on renal replacement therapy in both groups; all patients were receiving at least 2 inotropes with inotropic scores >10. Notably, the mean preoperative ECLS support duration was 7 (SD 6) vs 7 (SD 6) days in patients with or without CPB, respectively (*P* = 0.984).

**Table 1:** Preoperative characteristics of the overall population

Parameter	CPB (*n* = 198)	ECLS (*n* = 204)	SMD	*P*-value
Age, years (SD)	52 (11)	52 (11)	0.03	0.75
Male, *n* (%)	169 (85)	158 (77)	0.20	0.04
BMI, mean (SD)	26.8 (5.5)	26.3 (5.2)	0.09	0.37
Reoperative surgery, *n* (%)	53 (27)	42 (21)	0.15	0.14
ICM, *n* (%)	111 (56)	123 (60)	0.09	0.42
A fib, *n* (%)	80 (42)	69 (36)	0.05	0.65
Diabetes mellitus, *n* (%)	47 (24)	51 (25)	0.03	0.76
Peripheral artery disease, *n* (%)	10 (5)	14 (7)	0.08	0.44
CPR, *n* (%)	62 (31)	63 (31)	<0.01	0.90
Platelet count, ×10^3^, mean (SD)	119 (92)	86 (43)	0.51	<0.01
Haemoglobin, g/dl, mean (SD)	9.8 (1.6)	9.6 (1.6)	0.10	0.36
Lactate, mean (SD)	1.6 (1.4)	1.4 (1.7)	0.13	0.25
pH, mean (SD)	7.38 (0.12)	7.37 (0.11)	0.13	0.37
Liver enzymes/AST, mean (SD)	469 (1518)	428 (1020)	0.03	0.74
Liver enzymes/ALT, mean (SD)	302 (683)	365 (694)	0.10	0.45
Bilirubin, mean (SD)	3.0 (3.4)	3.2 (3.7)	0.04	0.68
CRP mg/l, mean (SD)	45.5 (75)	13.1 (8.8)	0.78	<0.01
Leucocytes, mean (SD)	12.4 (5)	12.4 (5.4)	0.02	0.89
INR, mean (SD)	1.4 (0.4)	1.5 (0.7)	0.24	0.03
Creatinine mg/dl, mean (SD)	1.9 (5.3)	1.5 (1)	0.14	0.26
Preoperative renal replacement therapy, *n* (%)	66 (32)	56 (27)	0.10	0.28
MELD score, mean (SD)	19 (7)	18 (8)	0.06	0.56
Preoperative intra-aortic balloon pump, *n* (%)	57 (29)	37 (18)	0.28	0.01
Preoperative ECMO cannulation (central), *n* (%)	30 (15)	19 (9)	0.18	0.09

A fib: atrial fibrillation; ALT: alanine transaminase; AST: aspartate aminotransferase; BMI: body mass index; CPB: cardiopulmonary bypass; CPR: cardiopulmonary resuscitation; CRP: C-reactive protein; ECLS: extracorporeal life support; ECMO: extracorporeal membrane oxygenation; ICM: ischaemic cardiomyopathy; INR: international normalized ratio; MELD: model of end-stage liver disease; SD: standard deviation; SMD: standardized mean difference.

**Table 2:** Preoperative characteristics: propensity matched population

Parameter	CPB	ECLS	SMD	*P*-value
	(*n* = 175)	(*n* = 175)		
Age, years, mean (SD)	53 (11)	52 (11)	0.02	0.88
Male, *n* (%)	149 (85)	149 (85)	<0.01	1.00
BMI, mean (SD)	27 (5.6)	26.4 (5.2)	0.11	0.31
Reoperative surgery, *n* (%)	48 (27)	39 (22)	0.12	0.26
ICM, *n* (%)	98 (56)	105 (60)	0.08	0.52
A Fib, *n* (%)	72 (43)	64 (39)	0.03	0.78
Diabetes, *n* (%)	45 (26)	46 (26)	0.01	0.90
Peripheral artery disease, *n* (%)	9 (5)	12 (7)	0.07	0.50
CPR, *n* (%)	53 (30)	55 (31)	0.03	0.81
Platelet count/×10^3^, mean (SD)	99 (56)	98 (53)	0.02	0.86
Haemoglobin g/dl, mean (SD)	9.8 (1.5)	9.6 (1.5)	0.11	0.32
Lactates, mean (SD)	1.5 (1.3)	1.5 (1.7)	0.01	0.93
pH, mean (SD)	7.37 (0.12)	7.38 (0.1)	0.10	0.40
Liver enzymes/AST, mean (SD)	487 (1592)	411 (957)	0.06	0.58
Liver enzymes/ALT, mean (SD)	321 (728)	358 (682)	0.06	0.68
Bilirubin, mean (SD)	3.2 (3.5)	3.3 (3.8)	0.02	0.82
CRP mg/l, mean (SD)	15.3 (10.5)	14.5 (11.1)	0.08	0.45
Leukocytes, mean (SD)	12 (4.8)	12.5 (5.1)	0.10	0.31
INR, mean (SD)	1.4 (0.4)	1.5 (0.7)	0.20	0.07
Creatinine mg/dl, mean (SD)	1.9 (5.3)	1.5 (1)	0.11	0.26
Preop renal replacement therapy, *n* (%)	61 (35)	50 (29)	0.14	0.20
MELD score, mean (SD)	19 (7)	18 (8)	0.07	0.52
Preoperative intra-aortic balloon pump, *n* (%)	47 (27)	35 (20)	0.16	0.13
Preoperative ECMO cannulation (central; *N*, %)	26 (15)	16 (9)	0.18	0.14

A fib: atrial fibrillation; ALT: alanine transaminase; AST: aspartate aminotransferase; BMI: body mass index; CPB: cardiopulmonary bypass; CPR: cardiopulmonary resuscitation; CRP: C-reactive protein; ECLS: extracorporeal life support; ECMO: extracorporeal membrane oxygenation; ICM: ischaemic cardiomyopathy; INR: international normalized ratio; MELD: model of end-stage liver disease; SD: standard deviation; SMD: standardized mean difference.

### Operative data

All patients received an implantable continuous flow LVAD, with apical cannula and ascending aorta outflow graft anastomosis. Devices used are reported in Table 3. As many as 38 patients had a less invasive approach via an anterolateral left thoracotomy and an upper hemisternotomy (6% in the CPB vs 16% in the ECLS group, respectively; *P* = 0.002). The total operating time was significantly longer in the CPB group [285 (SD 72) vs 209 (SD 75) min; *P* ≤ 0.001].

**Table 3:** Intra-/postoperative results: propensity matched cohort

Parameter	CPB	ECLS	*P*-value
	*N* = 175	*N* = 175	
Intraoperative, *n* (%)			
HeartWare HVAD	121 (69)	156 (89)	<0.001
HeartMate II	35 (20)	11 (6)	<0.001
HeartMate III	18 (10)	8 (5)	0.04
Other LVAD	1 (1)	0 (0)	–
Less-invasive operations, *n* (%)	10 (6)	28 (16)	0.002
CPB time, min, mean (SD)	106 (61)	–	–
Total operative time, min, mean (SD)	285 (72)	209 (75)	<0.001
Postoperative			
Chest tube output in 24 h, ml, mean (SD)	1513 (1311)	1390 (1121)	0.38
RVAD implantation, *n* (%)	87 (50)	71 (41)	0.08
RVAD duration days, mean (SD)	17 (13)	16 (13)	0.59
FFP units, day of surgery, mean (SD)	6.6 (7)	5.1 (5)	0.03
PRBC units, day of surgery, mean (SD)	8.2 (8)	6.5 (4)	0.01
PLT units, day of surgery, mean (SD)	3.3 (3)	3 (2)	0.38
Reexploration (bleeding), *n* (%)	72 (41)	51 (29)	0.01
Stroke, any (*N*, EPPY), *n* (%)	42 (0.18)	59 (0.18)	0.99
Disabling stroke, *n* (%)	13 (7)	15 (9)	0.69
Driveline infection, *n* (%)	37 (21)	56 (32)	0.02
GI bleeding, *n* (%)	28 (16)	29 (17)	0.88
Postoperative dialysis, *n* (%)	113 (66)	101 (58)	0.11
Pump thrombosis, *n* (%)	25 (14)	15 (9)	0.09
Outcome			
30-Day mortality, *n* (%)	47 (27)	36 (20)	0.20
Heart transplant, *n* (%)	38 (19)	36 (14)	0.79

CPB: cardiopulmonary bypass; ECLS: extracorporeal life support; EPPY: events per patient year; FFP: fresh frozen plasma; GI: gastrointestinal; LVAD: left ventricular assist device; PLT: platelet units; PRBC: packed red blood cells; RVAD: right ventricular assist device.

### Perioperative outcomes

The postoperative chest tube blood loss on the day of surgery was comparable between the groups [1513 (SD 1311) vs 1390 (SD 1121) ml; *P* = 0.3]; however, patients in the CPB group had a significantly greater need of reoperation for bleeding (41% vs 29%; *P* = 0.01) and also required a significantly greater number of packed red blood cells [8 (SD 8) vs 6 (SD 4) units; *P* = 0.001] and fresh frozen plasma [6 (SD 7) units vs 5 (SD 5) units; *P* = 0.03] compared to patients operated on while on ECLS. The number of platelets transfused was comparable. It was necessary to implant a temporary postoperative right ventricular assist device in 50% versus 41% of the patients with or without CPB, respectively (*P* = 0.08). No significant difference was observed for postoperative renal, liver and respiratory failure. The overall stroke rate was not significantly different between groups (CPB 42 events: total LVAD support duration 83 351 days, events per patient year 0.18 vs ECLS 59 events: total LVAD support duration 117 877 days, events per patient year 0.18; *P* = 0.99). Moreover, the incidence of stroke at 30 days did not different according to the pump type (HeartMate II 9.6% vs HVAD 7.2%; *P* = 0.5). Similarly, there was no statistically significant difference in the 30-day stroke rate in the CPB versus the ECLS group (CPB 5% vs ECLS 10%; *P* = 0.07). A total of 81 patients died in the hospital: 47 (27%), in the CPB versus 36 (20%) in the ECLS group (*P* = 0.2). Further, there was a statistically significant difference in the median LVAD support duration between the groups (ECLS = 366 days vs CPB = 256 days; *P* = 0.005).

**Figure 1:**
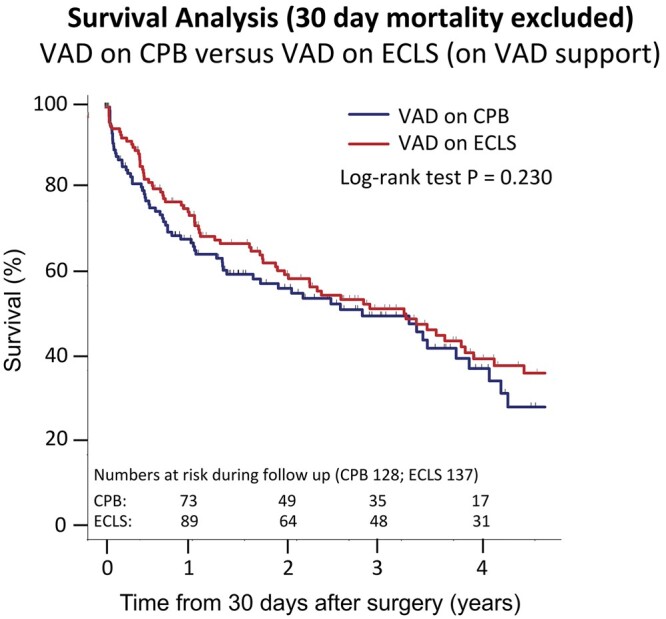
Kaplan–Meier survival curve of the study populations.

Follow-up was available for all survivors: Estimated survival for the overall cohort was 68% at 1 year and 32% at 5 years. When stratified by extracorporeal circulation technique, survival at 1 year was not statistically different between the 2 groups (65% vs 72%; *P* = 0.096); Kaplan–Meier survival did not show any significant difference in survival at 4.5 years (log-rank, *P* = 0.2) (Fig. 1).

## DISCUSSION

In this large, multicentre ‘real-world’ experience of patients having durable MCS implants while on ECLS for cardiogenic shock and failure to wean, data demonstrate similar survival rates for patients operated on with CPB or on ECLS as an extracorporeal circulation technique; however, omitting CPB is associated with a lower rate of re-exploration for bleeding and the need for blood products.

For several years, the standard extracorporeal circulation strategy of implanting an LVAD has involved CPB and a full sternotomy approach. However, less invasive techniques, even off-pump, are now becoming increasingly popular because they appear to be associated with reduction of surgical trauma, blood loss and hospital stay. Thus, avoidance of CPB in such a critically ill patient population is potentially an adjunct to decrease morbidity and mortality. There is a tremendous difference in the characteristics and functional status of patients who had an LVAD implanted during ECLS support. These patients are sicker, a majority of them have had cardiopulmonary resuscitation and are on a ventilator with respiratory dysfunction and right ventricular involvement; most importantly, a severe tendency to bleed secondary to ECLS-related coagulopathy is always present.

Despite the variability of implant strategies and the retrospective nature of this study, overall reported results in a propensity matched population show that implanting an LVAD and omitting CPB compare favourably to the standard surgical approach in terms of bleeding outcomes and are not associated with a higher rate of stroke. Our comparative analysis did not show significant differences in perioperative morbidity and early and late survival. Indeed, our results confirm that implanting a durable MCS in patients with prior ECLS support is still plagued by high morbidity and mortality. Estimated 30-day and long-term survival was comparable between the 2 groups, and the surgical technique was not associated with deaths.

This study reports an overall comparable adverse events profile in the largest cohort of patients in this clinical scenario; interestingly, bleeding complications were significantly higher in the CPB group. These results are in line with the pathophysiological background of CPB compared to ECLS and the role of activation of coagulation via other mechanisms besides contact with foreign surfaces. Interestingly, there was a higher rate of driveline infection in the ECLS group. The only explanation for this finding might be the fact that the ECLS group had a statistically longer ventricular assist device support duration than the CPB group (366 days vs CPB 256 days, *P* = 0.005).

Indeed, CPB has unique features inducing a systemic inflammation state [7], in some aspects different compared to ECLS. The presence of a blood–air interface due to cardiotomy suction and venous reservoirs has been associated with a higher degree of pro-inflammatory cytokines release and lower levels of anti-inflammatory cytokines such as IL-10 [8]. Haemodilution, moreover, is greater in CPB and is able *per se* to lead to increased neutrophil activation and systemic inflammatory response syndrome [9].

Theoretically, the higher degree of inflammation observed in CPB could induce a more pronounced activation of the coagulation system, leading to consumption coagulopathy, through the well-documented effects on fibrinolysis, platelet sequestration and degradation of coagulation factors.

Therefore, implanting an LVAD without CPB could potentially reduce the burden of these complications, leading to an improved postoperative course. Indeed, our study showed a significant advantage of the need for surgical re-exploration and the need for blood products; however, this situation did not turn into improved survival.

In a previous study, Saeed *et al.* showed a significant reduction of blood loss and of the need for blood products in a series of patients who were implanted with the HVAD in a similar scenario (15 vs 15 propensity matched CPB vs no CPB patients) [5]. The authors concluded that implanting an LVAD in patients on ECLS is feasible and has the advantage of minimizing additional blood trauma induced by the CPB circuit. It has been well documented that higher morbidity and mortality are correlated with the number of red blood cell transfusions in cardiac surgery.

Interestingly, that fact that less bleeding and fewer reexplorations were observed in the ECLS group did not translate into any outcome benefits. The groups in this study were well matched at baseline. However, there were more less invasive operations in the venoarterial-extracorporeal membrane oxygenation (ECMO) group, which might have explained the differences in the quantities of blood products needed. Less invasive surgery has been shown to reduce bleeding rates in LVAD candidates [10].

Notably, there were also significant differences in the pump type after matching: There were more HeartWare HVAD pumps implanted in the ECLS group. We do not believe that differences in the bleeding tendencies and blood product requirements are related to the type of pump [2].

New preliminary strategies, which apply the bridge-to-bridge concept via the axillary Impella 5.0, have provided excellent results in this patient population, and future studies are warranted to validate this approach [11]. This approach has been called the LVAD test because it not only allows for ECLS weaning but also for thorough evaluation of the right ventricle and optimization of the extracardiac conditions. In this light, we foresee a potential new tool to give these very sick patients the opportunity for a successful LVAD therapy.

### Limitations

This study was retrospective and non-randomized. To achieve an acceptable comparison basis with regard to the patient characteristics between the groups, we also included variables to the PSM that are a snapshot of a particular moment in time and may therefore not necessarily represent reproducible differences between the groups. Nevertheless, we opted for including these variables in the model as surrogate clinical parameters with a potential impact on postoperative outcome (e.g. platelet count). Yet, we have to acknowledge that for some parameters (e.g. intra-aortic balloon pump, central ECMO, renal replacement therapy), the differences between both groups are more important than usually required for PSM.

The decision to proceed to CPB was left to the preference of the surgeon; presumably, there might be institutional and surgeon bias across the 11 centres as to which technique is selected. However, it is important to acknowledge that not all potential LVAD candidates are suitable for the ECLS approach; indeed, that strategy, if systematically applied, can jeopardize the benefits of LVAD therapy by omitting a necessary concomitant procedure or neglecting thorough exploration of the LV cavity for thrombi. All patients were screened for LV thrombi using echocardiography at the time of surgery, and CPB was always used if thrombi were detected. Unfortunately, the presence of thrombi was not documented in the study. Notably, based on our clinical experience, the presence of thrombi in ECMO patients with full anticoagulation is rare. Some patients were operated on with a less invasive technique: It was assumed that this approach might reduce perioperative bleeding; however, the results are still inconclusive and have focused on elective patients rather than on those described in our study. Our population received a variety of devices, which might be relevant in terms of duration of surgery and bleeding risk because contemporary centrifugal devices have been miniaturized and do not require a pump pocket; therefore, they cause surgical trauma. However, transfusion protocols differ across centres, which might account for the differences reported in the utilization of blood product. Indeed, surgical re-exploration is a strong end-point and cannot be disregarded as significant. There were more HeartMate II devices in the CPB group. However, the number of HeartMate 3 pumps in the overall population is small. Therefore, these results do not describe the current device selection in the real world. Further, some follow-up data, for instance, the types and timing of postoperative stroke (ischaemic versus haemorrhagic) are currently not available to report in this manuscript.

## CONCLUSION 

In conclusion, an LVAD implant directly from ECLS is a challenging procedure, and any effort that would improve the results is to be acknowledged. In this regard, the surgical strategy without conversion to CPB, in selected cases, is safe and associated with significantly better results in terms of perioperative bleeding; however, this benefit does not have a major benefit for major clinical outcomes. Future randomized controlled studies are warranted.

**Conflict of interest:** none declared.

### Data Availability Statement

All the data are contained in the manuscript.

### Author contributions

**Federico Pappalardo:** Conceptualization; Writing—original draft; Writing—review & editing. **Evgenij Potapov:** Conceptualization; Investigation; Writing—review & editing. **Antonio Loforte:** Conceptualization; Investigation; Writing—review & editing. **Michiel Morshuis:** Data curation; Investigation. **David Schibilsky:** Data curation; Investigation. **Daniel Zimpfer:** Conceptualization; Data curation; Investigation. **Julia Riebandt:** Data curation; Investigation. **Christian Etz:** Data curation; Investigation. **Matteo Attisani:** Data curation; Investigation. **Mauro Rinaldi:** Data curation; Investigation. **Assad Haneya:** Data curation; Investigation. **Faiz Ramjankhan:** Data curation; Investigation. **Dirk W Donker:** Data curation; Investigation. **Ulrich P Jorde:** Supervision; Writing—review & editing. **Daniel Lewin:** Data curation; Investigation. **Radi Wieloch:** Data curation; Investigation. **Rafael Ayala:** Data curation; Investigation. **Jochen Cremer:** Data curation; Investigation. **Letizia Bertoldi:** Formal analysis; Methodology. **Michael Borger:** Data curation; Investigation. **Artur Lichtenberg:** Data curation; Investigation. **Jan Gummert:** Data curation; Investigation. **Diyar Saeed:** Conceptualization; Data curation; Investigation; Methodology; Writing—review & editing.

### Reviewer information

Interactive CardioVascular and Thoracic Surgery thanks Davide Pacini, Piergiorgio Tozzi and the other, anonymous reviewer(s) for their contribution to the peer review process of this article.

## ABBREVIATIONS

CPB: Cardiopulmonary bypass
ECLS: Extracorporeal life support
ECMO: Extracorporeal membrane oxygenation
LVAD: Left ventricular assist device
MCS: Mechanical circulatory support
PSM: Propensity score matching
SD: Standard deviation
